# New Medical Work in Paris

**Published:** 1862-07

**Authors:** 


New Medical Work in Paris.—An important work, the "Bibliographie Universelle de la Medecine, de la Chirurgie, et de la Pharmacie Militaire," has been undertaken by M. Rozier, the editor, and tome 1st has already appeared. The circle embraced by this new compilation is one of great width; its value will mainly consist in rescuing from oblivion many interesting monographs already set aside and forgotten by our ungrateful and proficient age, and in furnishing the student with a complete list of the authors who have treated any particular subject which he may desire to investigate.—*Ibid.*

